# Inflammatory Stress Induced by Intraperitoneal Injection of LPS Increases Phoenixin Expression and Activity in Distinct Rat Brain Nuclei

**DOI:** 10.3390/brainsci12020135

**Published:** 2022-01-20

**Authors:** Tiemo Friedrich, Martha Anna Schalla, Miriam Goebel-Stengel, Peter Kobelt, Matthias Rose, Andreas Stengel

**Affiliations:** 1 Charité Center for Internal Medicine and Dermatology, Department for Psychosomatic Medicine, Charite–Universitätsmedizin Berlin, Corporate Member of Freie Universität Berlin, Humboldt-Universität zu Berlin and Berlin Institute of Health, 12203 Berlin, Germany; tiemo.friedrich@charite.de (T.F.); martha.schalla@charite.de (M.A.S.); miriam.goebel-stengel@gmx.de (M.G.-S.); peter.kobelt@charite.de (P.K.); matthias.rose@charite.de (M.R.); 2 Department of Internal Medicine, Helios Kliniken GmbH, 78628 Rottweil, Germany; 3 Department of Psychosomatic Medicine and Psychotherapy, University Hospital Tübingen, 72076 Tübingen, Germany

**Keywords:** brain–gut axis, c-Fos, inflammation, lipopolysaccharide, stress, vasopressin

## Abstract

Due to phoenixin’s role in restraint stress and glucocorticoid stress, as well as its recently shown effects on the inflammasome, we aimed to investigate the effects of lipopolysaccharide (LPS)-induced inflammatory stress on the activity of brain nuclei-expressing phoenixin. Male Sprague Dawley rats (*n* = 6/group) were intraperitoneally injected with either LPS or control (saline). Brains were processed for c-Fos and phoenixin immunohistochemistry and the resulting slides were evaluated using ImageJ software. c-Fos was counted and phoenixin was evaluated using densitometry. LPS stress significantly increased c-Fos expression in the central amygdaloid nucleus (CeM, 7.2-fold), supraoptic nucleus (SON, 34.8 ± 17.3 vs. 0.0 ± 0.0), arcuate nucleus (Arc, 4.9-fold), raphe pallidus (RPa, 5.1-fold), bed nucleus of the stria terminalis (BSt, 5.9-fold), dorsal motor nucleus of the vagus nerve (DMN, 89-fold), and medial part of the nucleus of the solitary tract (mNTS, 121-fold) compared to the control-injected group (*p* < 0.05). Phoenixin expression also significantly increased in the CeM (1.2-fold), SON (1.5-fold), RPa (1.3-fold), DMN (1.3-fold), and mNTS (1.9-fold, *p* < 0.05), leading to a positive correlation between c-Fos and phoenixin in the RPa, BSt, and mNTS (*p* < 0.05). In conclusion, LPS stress induces a significant increase in activity in phoenixin immunoreactive brain nuclei that is distinctively different from restraint stress.

## 1. Introduction

In recent years, interest in phoenixin, with its 14 and 20 amino acid sequences, has continuously increased and its functions in basic physiological processes have become more and more apparent. Phoenixin was first described in 2013 as a peptide involved in reproduction [1], but has since been shown to be involved in multiple processes such as pain sensitivity [2], pruritus [3], inhibition of anxiety [4,5], increased food intake [6,7], influence of vasopressin secretion [8], as well as stress mediation [9]. Although not definitively confirmed, GPR173 is widely accepted as a putative phoenixin receptor [10].

In our previous work, we studied phoenixin’s role in food intake as well as anxiety and stress reactions. Phoenixin’s effects of increasing food intake after intracerebroventricular (ICV) injection correlated with a distinct increase in activity in nesfatin-1 positive neurons in several brain nuclei, giving rise to speculatively counterbalancing roles of these two peptides [6]. A similar counterbalancing effect was observed regarding anxiety, where phoenixin is negatively correlated with levels of anxiety [4,5], while nesfatin-1 shows a positive correlation with anxiety in women [11,12].

Our goal was to evaluate whether phoenixin could also play a role in response to inflammatory stress. Therefore, we analyzed brain nuclei, which were previously described to physiologically express phoenixin [13], after peripheral injection of the well-established inflammatory agent lipopolysaccharide (LPS, as an immune-triggering part of Gram-negative bacteria) [14] using a well-established marker for neuronal activity, c-Fos [15].

## 2. Materials and Methods

### 2.1. Animals

Adult male Sprague Dawley rats (Envigo, Düsseldorf, Germany) weighing 251–295 g were housed under standard conditions (12 h light/dark cycle, 6:00 a.m./p.m., 21–23 °C) in groups of 3 with ad libitum access to standard rodent chow (ssniff Spezialdiäten GmbH, Soest, Germany) and water. Male animals were chosen to avoid the estrous cycle as a confounding factor. After one week of acclimatization, animals were handled daily by the investigators to familiarize the animals to the investigators. Handling included simulated intraperitoneal (IP) injections employing a pointed pencil. In order to avoid a confounding stress reaction due to single housing after injection, animals were single housed for 4 h on two separate days during this period. These cages remained unaltered and were reused for the same animal in the experiments to house the animals in a familiar environment and avoid confounding stress due to unfamiliar cages. The experiments were conducted after approval by the state authority for animal research (G0132/16 approved on 8 August 2016 by Landesamt für Gesundheit und Soziales Berlin, LaGeSo Berlin) and followed all institutional ethics guidelines.

### 2.2. Intraperitoneal Injection of LPS

Animals remained in their group housing cages until IP injection with lipopolysaccharide (LPS, 250 μg/kg body weight in 300 μL sterile saline) or control (300 μL saline, *n* = 6/group) between 7:55 a.m. and 10:30 a.m. The group size was based on a previous publication [16]. Each cage housing three animals was randomly assigned to either the LPS or control group. The dose of LPS was based on a previous publication [17]. Injection took place in a separate room to avoid stress for other animals. The animals were housed in a single cage after the injection. After 180 min, the animals were anesthetized and sacrificed. The timing of finalization was chosen according to previous research [18]. During the 180 min period, fecal pellet output was monitored as a well-established surrogate of stress [19].

### 2.3. Anesthesia

To achieve sufficient anesthesia, animals were IP injected with 100 mg/kg body weight ketamine (Ketanest^TM^, Curamed, Karlsruhe, Germany) mixed with 10 mg/kg xylazine (Rompun^TM^ 2%, Bayer, Leverkusen, Germany) as previously described [20]. Depth of anesthesia was ensured via application of a standardized pain stimulus before the animal was sacrificed.

### 2.4. Brain Processing

Transcardial perfusion was performed as previously described [9]. After thoracotomy, the left heart ventricle was incised, and the perfusion cannula was inserted into the ascending aorta. The right ventricle was incised to allow drainage. After an initial flushing with saline for one minute, perfusion with picric acid fixation solution (4% paraformaldehyde + 14% saturated picric acid solved in 0.1 M phosphate buffered saline, PBS, adjusted to pH 7.4) was performed for 15 min. The animals were then decapitated, and the brains were carefully extracted to avoid damage. Post-fixation was performed by washing in picric acid fixation solution for 24 h followed by dehydration in 10% sucrose solution for 48 h. Afterwards, brains were snap-frozen using −80 °C 2-methylbutane (Carl Roth KG, Karlsruhe, Germany) and subsequently stored at −80 °C. The brains were cut into 25 μm coronal sections using a cryostat (CryoStar NX70, Thermo Fisher Scientific, Waltham, MA, USA) and subsequently stored at −20 °C in anti-freeze solution.

### 2.5. Immunostaining

Immunohistochemical double staining was performed as described in our previous publications [6,9,21] using the free-floating technique. Sections were incubated overnight in rabbit polyclonal anti-c-Fos antibody (1:20,000, Catalog No. ABE457, Merck Millipore, Darmstadt, Germany) at 4 °C. Incubation with a secondary goat anti-rabbit IgG Fab fragment (1:1000, Catalog No. 111-067-003, Jackson ImmunoResearch Laboratories Inc., West Grove, PA, USA) was performed for 2 h at room temperature. Initial staining was performed using diaminobenzidine tetrahydrochloride (DAB, Sigma-Aldrich, Darmstadt, Germany) with nickel ammonium sulfate (Fisher Scientific, Waltham, MA, USA) catalyzed by H_2_O_2_, leading to a dark blue/black staining of c-Fos-positive structures. Double labeling was achieved by incubating a primary rabbit anti-rat anti-phoenixin antibody (1:500, Catalog No. G-079-01, Phoenix Pharmaceuticals Inc., Burlingame, CA, USA) overnight, followed by a secondary biotinylated goat anti-rabbit IgG (1:1000 Catalog No. 111-065-144, Jackson ImmunoResearch) for 2 h at room temperature. Visualization was performed using DAB without nickel to achieve a brown staining of immunopositive structures. Every third slide was used for evaluation.

The primary phoenixin antibody binds to the phoenixin sequences of 5, 8, 14, and 20 amino acids [22]; therefore, the immunohistochemistry does not allow a differentiation between phoenixin 14 and 20. Specificity to phoenixin was previously tested by pre-absorption [13].

### 2.6. Slide Evaluation

Brain nuclei were localized using the standard coordinates based on the rat brain atlas [23]. Exact coordinates relative to bregma were BSt (Anterior/Posterior (AP) 0.84; Medial/Lateral (ML) 1.2–1.6; Dorsal/Ventral (DV) 6.6–7.4 to AP −0.48; ML 0.8–1.8; DV 6.0–6.8; bed nucleus of the stria terminalis), PVN (AP −1.08; ML 0–0.6; DV 6.8–7.6 to AP −1.92; ML 0.0–1.0; DV 7.8–8.8; paraventricular nucleus), SON (AP −0.72; ML 1.2–1.8; DV 9.2–9.4 to AP −1.56; ML 2.0–2.6; DV 9.2–9.6; supraoptic nucleus), CeM (AP −1.56; ML 3.2–3.8; DV 8.0–8.8 to AP −2.92; ML 4.0–4.4; DV 7.6–8.4 central amygdaloid nucleus, medial division), ARC (AP −1.72; ML 0.0–0.2; DV 9.6–10.0 to AP −3.24; ML 0.0–0.6; DV 9.4–10.2; arcuate nucleus), RPa (AP −9.72; ML 0.0–0.1; DV 10.4–10.8 to AP −12.12; ML 0.0–0.1; DV 10.4–10.8; nucleus raphe pallidus), DMN (AP −12.84; ML 0.8–1.2; DV 7.8–8.0 to AP −14.40; ML 0.1–0.8; DV 8.0–8.2; dorsal motor nucleus of the vagus nerve), and mNTS (AP −13.68; ML 0.2–0.8; DV 7.5–7.8 to AP −14.40; ML 0.2–0.6; DV 7.6–8.0; nucleus of the solitary tract, medial part). Evaluation of the slides was performed by an investigator blinded to treatment using ImageJ software (ImageJ 1.52a, National Institute of Health, Bethesda, MD, USA). A minimum of five representative photos of each nucleus and animal were taken using a light microscope (Zeiss Axiophot, Zeiss, Jena, Germany) and a connected camera (AxioCam HRc, Zeiss, Jena, Germany). Evaluation of c-Fos-positive nuclei was performed manually using ImageJ’s counting function. Phoenixin signal was measured using ImageJ’s “measure” function. Five areas of 100 × 100 pixels were measured per photo, each corrected for background noise by measuring two 400 × 400 pixel quadrants outside the nucleus and subtracting the mean result. Five slides were evaluated per animal and nucleus.

### 2.7. Statistical Analysis

All results were analyzed using SPSS 25 (IBM Corp. 2017, IBM SPSS Statistics for Windows, Version 25.0, Armonk, NY, USA). Normality was assessed using the Kolmogorov–Smirnov test. If normal distribution was confirmed, differences between groups were evaluated using the t-test; otherwise, the Mann–Whitney U-test was employed. Correlation analysis was performed using Pearson’s analysis. Data are expressed as mean ± SEM. Results were considered significant if *p* < 0.05 was calculated.

## 3. Results

**Figure 1:**
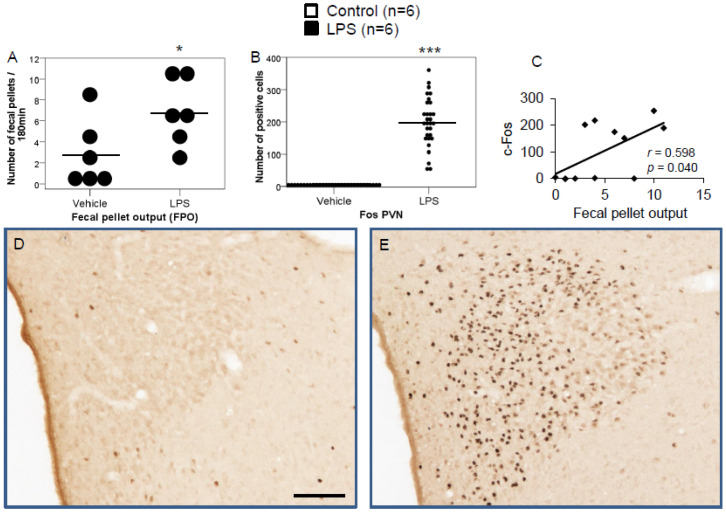
LPS significantly increases fecal pellet output (FPO) compared to control (**A**). LPS stimulates c-Fos expression in the paraventricular nucleus. Ad libitum-fed rats were subjected to IP injection of LPS or control, and 180 min after the injection, brains were processed for c-Fos and phoenixin double-immunohistochemistry. LPS stress significantly increased the number of c-Fos-positive cells compared to controls (**B**) and the number of c-Fos positively correlated with fecal pellet output (**C**). Representative pictures of the evaluated slides are shown in (**D**) (control) and (**E**) (LPS). The scale bar represents 100 µm in the overview. Data are expressed as individual data points (5/animal) and mean in (**A**,**B**). Mean values per animal are plotted in (**C**). * *p* < 0.05, *** *p* < 0.001.

**Figure 2:**
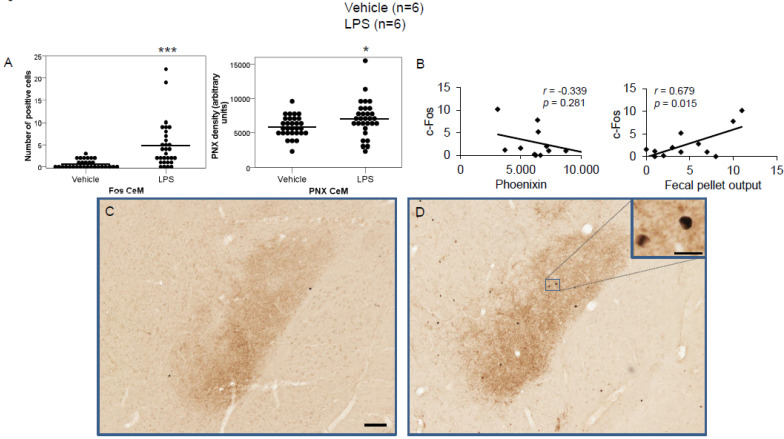
LPS stimulates c-Fos and phoenixin expression in the medial division of the central amygdaloid nucleus. Ad libitum-fed rats were subjected to IP injection of LPS or control, and 180 min after the injection, brains were processed for c-Fos and phoenixin double-immunohistochemistry. LPS stress significantly increased the number of c-Fos-positive cells and phoenixin density compared to controls (**A**). No significant correlation was observed between c-Fos and phoenixin immunoreactivity, while the number of c-Fos positively correlated with fecal pellet output (**B**). Representative pictures of the evaluated slides are shown in (**C**) (control) and (**D**) (LPS). The scale bar represents 100 µm in the overview and 20 µm in the insert. Data are expressed as individual data points (5/animal) and mean in (**A**). Mean values per animal are plotted in (**B**). * *p* < 0.05, *** *p* < 0.001. Abbreviation: CeM, central amygdaloid nucleus, medial division.

**Figure 3:**
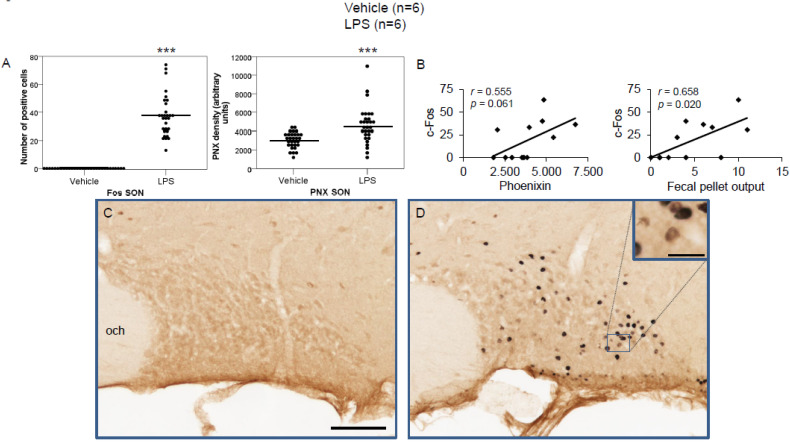
LPS stimulates c-Fos and phoenixin expression in the supraoptic nucleus. Ad libitum-fed rats were subjected to IP injection of LPS or control, and 180 min after the injection, brains were processed for c-Fos and phoenixin double-immunohistochemistry. LPS stress significantly increased the number of c-Fos-positive cells and phoenixin density compared to controls (**A**). No significant correlation was observed between c-Fos and phoenixin immunoreactivity, while the number of c-Fos positively correlated with fecal pellet output (**B**). Representative pictures of the evaluated slides are shown in (**C**) (control) and (**D**) (LPS). The scale bar represents 100 µm in the overview and 20 µm in the insert. Data are expressed as individual data points (5/animal) and mean in (**A**). Mean values per animal are plotted in (**B**). *** *p* < 0.001. Abbreviations: opt, optic tract; SON, supraoptic nucleus.

**Figure 4:**
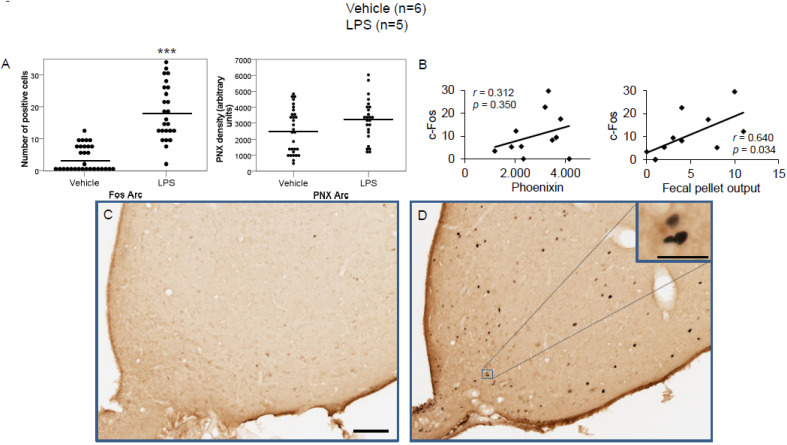
LPS stimulates c-Fos expression in the arcuate nucleus. Ad libitum-fed rats were subjected to IP injection of LPS or control, and 180 min after the injection, brains were processed for c-Fos and phoenixin double-immunohistochemistry. LPS stress significantly increased the number of c-Fos-positive cells but did not alter phoenixin density compared to controls (**A**). No significant correlation was observed between c-Fos and phoenixin immunoreactivity, while the number of c-Fos positively correlated with fecal pellet output (**B**). Representative pictures of the evaluated slides are shown in (**C**) (control) and (**D**) (LPS). The scale bar represents 100 µm in the overview and 20 µm in the insert. Data are expressed as individual data points (5/animal) and mean in (**A**). Mean values per animal are plotted in (**B**). *** *p* < 0.001. Abbreviations: 3V, third brain ventricle; Arc, arcuate nucleus.

**Figure 5:**
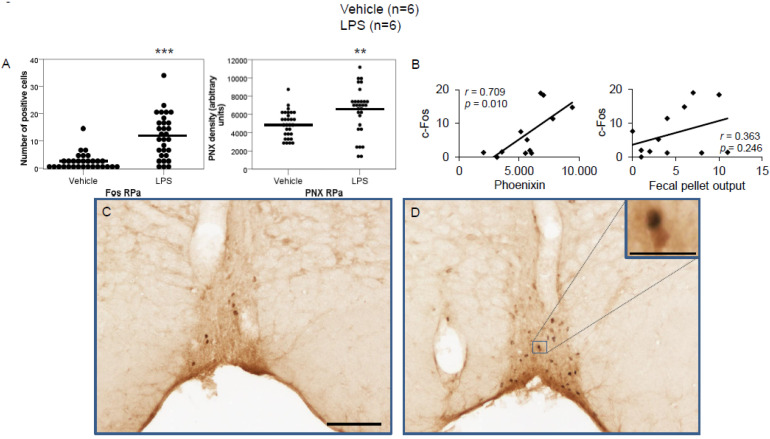
LPS stimulates c-Fos and phoenixin expression in the raphe pallidus. Ad libitum-fed rats were subjected to IP injection of LPS or control, and 180 min after the injection, brains were processed for c-Fos and phoenixin double-immunohistochemistry. LPS stress significantly increased the number of c-Fos-positive cells and phoenixin density compared to controls (**A**). A significant correlation was observed between c-Fos and phoenixin immunoreactivity, but not between the number of c-Fos and fecal pellet output (**B**). Representative pictures of the evaluated slides are shown in (**C**) (control) and (**D**) (LPS). The scale bar represents 100 µm in the overview and 20 µm in the insert. Data are expressed as individual data points (5/animal) and mean in (**A**). Mean values per animal are plotted in (**B**). ** *p* < 0.01, *** *p* < 0.001. Abbreviations: py, pyramidal tract; RPa, raphe pallidus.

**Figure 6:**
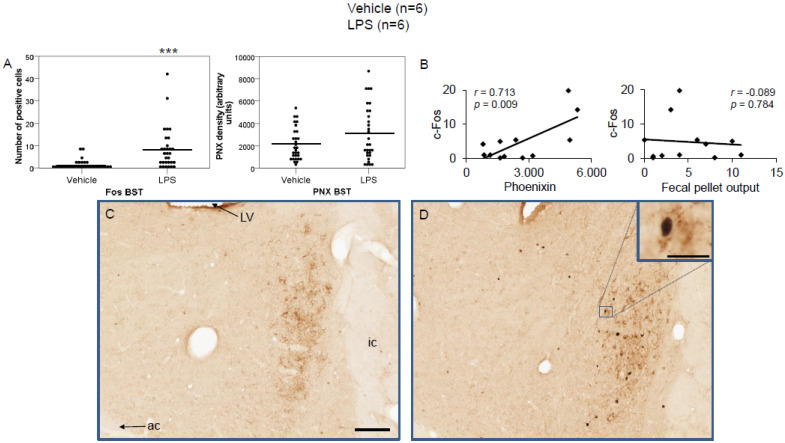
LPS stimulates c-Fos expression in the bed nucleus of the stria terminalis. Ad libitum-fed rats were subjected to IP injection of LPS or control, and 180 min after the injection, brains were processed for c-Fos and phoenixin double-immunohistochemistry. LPS stress significantly increased the number of c-Fos-positive cells but did not alter phoenixin density compared to controls (**A**). A significant correlation was observed between c-Fos and phoenixin immunoreactivity, but not between the number of c-Fos and fecal pellet output (**B**). Representative pictures of the evaluated slides are shown in (**C**) (control) and (**D**) (LPS). The scale bar represents 100 µm in the overview and 20 µm in the insert. Data are expressed as individual data points (5/animal) and mean in (**A**). Mean values per animal are plotted in (**B**). *** *p* < 0.001. Abbreviations: ac, anterior commissure; BSt, bed nucleus of the stria terminalis; ic, internal capsule; LV, lateral ventricle.

**Figure 7:**
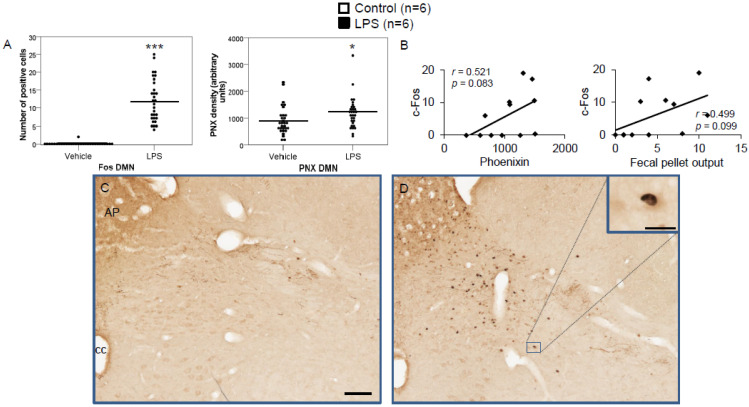
LPS stimulates c-Fos and phoenixin expression in the dorsal motor nucleus of the vagus nerve. Ad libitum-fed rats were subjected to IP injection of LPS or control, and 180 min after the injection, brains were processed for c-Fos and phoenixin double-immunohistochemistry. LPS stress significantly increased the number of c-Fos-positive cells and increased phoenixin expression in the DMN (**A**). A non-significant correlation was observed between c-Fos and phoenixin immunoreactivity in the DMN (**B**), as well as c-Fos and fecal pellet (**B**). Representative pictures of the evaluated slides are shown in (**C**) (control) and (**D**) (LPS). The scale bar represents 100 µm in the overview and 20 µm in the insert. Data are expressed as individual data points (5/animal) and mean in (**A**). Mean values per animal are plotted in (**B**). * *p* < 0.05 and *** *p* < 0.001. Abbreviations: AP, area postrema; cc, central canal.

**Figure 8:**
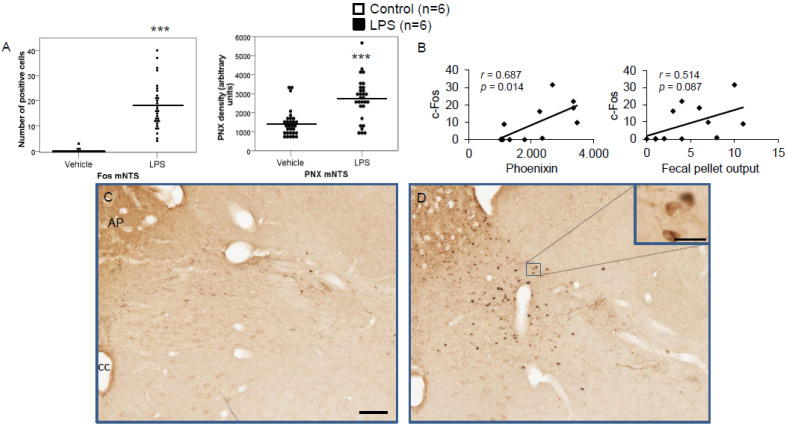
LPS stimulates c-Fos and phoenixin expression in the nucleus of the solitary tract. Ad libitum-fed rats were subjected to IP injection of LPS or control, and 180 min after the injection, brains were processed for c-Fos and phoenixin double-immunohistochemistry. LPS stress significantly increased the number of c-Fos-positive cells and increased phoenixin expression in the NTS (**A**). A significant correlation was observed between c-Fos and phoenixin immunoreactivity in the NTS (**A**), while the correlation between the number of c-Fos-positive neurons and fecal pellet output was non-significant. Representative pictures of the evaluated slides are shown in (**C**) (control) and (**D**) (LPS). The scale bar represents 100 µm in the overview and 20 µm in the insert. Data are expressed as individual data points (5/animal) and mean in (**A**). Mean values per animal are plotted in (**B**). *** *p* < 0.001. Abbreviations: AP, area postrema; cc, central canal.

We previously studied the physiological expression of phoenixin in undisturbed rats [13]; therefore, in this study, we focused on brain nuclei containing phoenixin immunoreactive neurons. We thus evaluated the central nucleus of the amygdala (CeM), supraoptic nucleus (SON), arcuate nucleus (Arc), raphe pallidus (RPa), bed nucleus of the stria terminals (BSt), dorsal motor nucleus of the vagus nerve (DMN), and the medial part of the nucleus of the solitary tract (mNTS), as well as the paraventricular nucleus (PVN) as a validation of our stress model. Representative images of the evaluated slides are shown in the figures (Figure 1D,E, Figure 2C,D, Figure 3C,D, Figure 4C,D, Figure 5C,D, Figure 6C,D, Figure 7C,D and Figure 8C,D).

### 3.1. Peripheral Inflammatory Stress Increases the Number of C-Fos in Several Brain Nuclei in Rats

Peripheral IP injection of LPS led to a significant increase in fecal pellet output (FPO) compared to the output of control rats (*p* < 0.05; Figure 1A). Moreover, LPS increased the number of c-Fos-positive neurons in all evaluated nuclei. A robust stress response was validated through highly significant c-Fos reactivity in the PVN compared to control (198.9 ± 14.6 vs. 1.4 ± 0.9, *p* < 0.001; Figure 1B), which correlated with FPO (Figure 1C).

LPS injection induced a significant increase in the number of c-Fos-positive neurons in the CeM (4.83 ± 0.96 vs. 0.67 ± 0.17; *p* < 0.001; Figure 2A), SON (37.5 ± 2.75 vs. 0.0 ± 0.0; *p* < 0.001; Figure 3A), Arc (18.24 ± 1.75 vs. 3.7 ± 0.79; *p* < 0.001; Figure 4A), RPa (11.7 ± 1.47 vs. 2.27 ± 0.57; *p* < 0.001; Figure 5A), BSt (8.27 ± 1.74 vs. 1.37 ± 0.42; *p* < 0.001; Figure 6A), DMN (12.07 ± 1.1 vs. 0.07 ± 0.07; *p* < 0.001; Figure 7A), and mNTS (17.77 ± 1.66 vs. 0.17 ± 0.11; *p* < 0.001; Figure 8A) compared to the control group. 

### 3.2. Peripheral Inflammatory Stress Increases Phoenixin Immunoreactivity in Several Brain Nuclei in Rats

Although our previously used semi-quantitative scoring system highly correlated with densitometric results [9], in the present study, we exclusively focused on densitometric evaluation to avoid any possible bias.

Densitometry showed a significant increase in phoenixin density in the CeM (7044.8 ± 490.7 vs. 5843.2 ± 271.4; *p* = 0.04; Figure 2A), SON (4637.0 ± 361.7 vs. 3054.8 ± 151.7; *p* < 0.001; Figure 3A), RPa (6463.7 ± 464.7 vs. 4895.5 ± 263.9; *p* < 0.01; Figure 5A), DMN (1181.65 ± 107.6 vs. 908.3 ± 95.5; *p* = 0.03; Figure 7A), and mNTS (2712.1 ± 203.3 vs. 1436.0 ± 128.4; *p* < 0.001; Figure 8A), while missing a statistically significant difference in the Arc (1.3-fold; *p* = 0.08; Figure 4A) and BSt (1.4-fold, *p* = 0.08; Figure 6A). 

Correlation analyses showed a significant positive correlation between c-Fos activity and phoenixin immunoreactivity in the RPa (*r* = 0.709, *p* = 0.010; Figure 5B), BSt (*r* = 0.713, *p* = 0.009; Figure 6B), and mNTS (*r* = 0.687, *p* = 0.014; Figure 8B), while narrowly missing significance in the SON (*r* = 0.555, *p* = 0.061; Figure 3B) and DMN (*r* = 0.521, *p* = 0.083; Figure 7B). No correlations between the number of c-Fos-positive cells and phoenixin immunoreactivity were observed in the CeM (*r* = −0.339, *p* = 0.281; Figure 2B) and Arc (*r* = 0.321, *p* = 0.350; Figure 4B).

Lastly, fecal pellet output correlated with the number of c-Fos-positive cells in the CeM (*r* = 0.679, *p* = 0.015; Figure 2B), SON (*r* = 0.679, *p* = 0.015; Figure 3B), and Arc (*r* = 0.640, *p* = 0.034; Figure 4B).

## 4. Discussion

After our initial paper, which evaluated the involvement of phoenixin in reaction to the emotional stressor restraint [9], the goal of this study was to assess whether this distinct change in neuronal activity and immunoreactivity would also be present after an immunological stressor. In order to evaluate the effects of LPS-induced stress on the neuronal activity in phoenixin immunoreactive nuclei, we used LPS injection as an immunological stressor to evaluate the histological response. Our results showed a significant LPS-induced increase in the number of c-Fos-positive neurons in all evaluated nuclei, namely the CeM, SON, Arc, RPa, BSt, DMN, and mNTS. The PVN, well known to respond to immunological stress [17], also showed an increased number of c-Fos-positive cells after LPS injection. The effectiveness of the stressor is further corroborated by increased fecal pellet output in LPS-treated rats, reflecting increased activation of the hypothalamus–pituitary–adrenal axis and activation of gut corticotropin-releasing factor signaling [24].

Phoenixin immunoreactivity was significantly increased in the CeM, SON, RPa, DMN, and mNTS. Our previous study evaluating restraint stress showed increased phoenixin expression in the RPa, DMN, and mNTS [9], giving rise to—although overlaps exist—a stressor-specific reaction. A significant correlation between the number of c-Fos-positive cells and phoenixin immunoreactivity was observed in the RPa, mNTS, and interestingly, also in the BSt, although levels of phoenixin expression were not significantly elevated in the BSt. These findings support the hypothesis that phoenixin expression correlates with the magnitude of the stress response and suggest that an increase in phoenixin expression in the mNTS is a more general stress-related response triggered by several stressors, while other nuclei are likely activated by stress-specific triggers.

The amygdala and the SON showed significantly higher c-Fos immunoreactivity after LPS stress, which did not occur after restraint stress. These findings are in line with results from previous studies, which found a much higher activation of the SON after LPS compared to restraint stress, as well as an increase in neuronal activity in the SON after osmotic stimulation by an IP injection of 9% NaCl [25].

**Figure 9:**
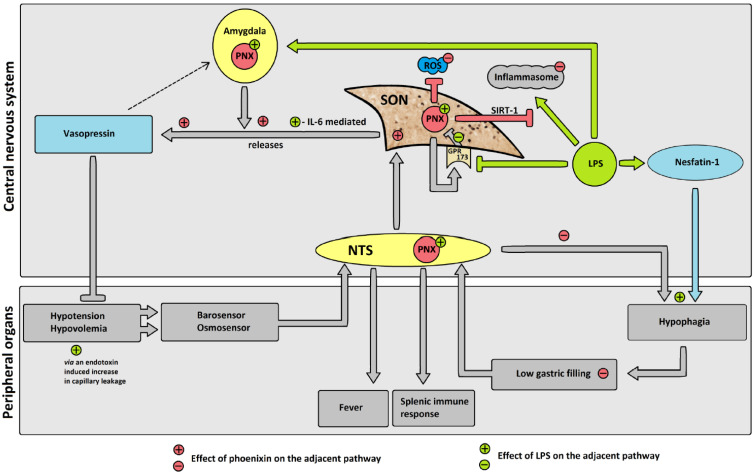
Hypothesized effects of LPS on phoenixin (PNX) and possible subsequent pathways. Gray pathways: proposed physiological pathway. Green pathways: proposed effects of LPS. Red pathways: proposed changes due to PNX. Adjacent +/− in respective color: influence of LPS/PNX on physiological pathway. LPS reduces GPR173 expression, thereby decreasing the negative feedback of GPR173 on PNX expression, leading to an increase in phoenixin expression. Increased PNX leads to an increase in vasopressin secretion. Inflammasome activation due to LPS is reduced by PNX via the SIRT-1 pathway. PNX reduces ROS. PNX induces eating (i.e., reduces hypophagia) and thereby increases gastric filling. Abbreviations: G-protein coupled receptor 173 (GPR173); interleukin-6 (IL-6); lipopolysaccharide (LPS); nucleus of the solitary tract (NTS); phoenixin (PNX); reactive oxygen species (ROS); sirtuin-1 pathway (SIRT-1); supraoptic nucleus (SON).

Previous studies showed an increase in vasopressin expression in the SON after osmotic stimulation after LPS injection [26]. Phoenixin was shown to increase the secretion of vasopressin in vitro in neurons from the SON, as well as to increase plasma vasopressin levels after ICV injection [8]. We previously detected an increase in drinking behavior after ICV injection of phoenixin [6], which coincided with a significant increase in c-Fos activity in the SON. Vasopressin-producing cells in the SON receive signals from osmoreceptors [27] as well as baroreceptors transmitted via the NTS [28,29], thereby influencing thirst and renal retention [27]. Overall, the increased phoenixin expression in the SON after LPS-induced immunological stress could be a physiological reaction to avoid septic hypovolemia (Figure 9). This would also fall in line with the observed reduction in GPR173 expression by LPS [30], since GPR173—the putative phoenixin receptor—is highly expressed in the SON [10] and could proposedly be part of a negative feedback loop with phoenixin. Phoenixin could, therefore, speculatively act as an “osmo-sensitizer” in the SON under conditions of stress, increasing the effects of afferent signals reaching the SON.

Although we did not find a significant increase in phoenixin expression in the BSt, phoenixin expression significantly correlated with c-Fos activity in this nucleus. The BSt receives afferent signals from limbic areas and has efferent projections to the SON [28]. It can trigger an acute vasopressin release, as shown by microinjection of carbachol in the BSt, whose effect was ameliorated by pretreatment of the SON with a neurotransmission blocker [31].

This proposed mode of action of phoenixin as a homeostasis regulating peptide under conditions of inflammation would fall in line with recently shown anti-inflammatory properties of phoenixin itself. Studies on phoenixin’s influence on the LPS-induced activation of the inflammasome in microglia [32] and astrocytes [30] in vitro recently showed robust anti-inflammatory effects. These effects were attributed to a reduction in reactive oxygen species and an increase in superoxide dismutase activity [32], as well as a reduction in NLRP3 inflammasome activity and the eIF-2α/ATF4/CHOP/GADD34 pathway, leading to a reduction in neuroinflammation [30]. The expression of phoenixin’s putative receptor GPR173 [10] was also reduced after LPS-induced stress [30]. We observed an increase in phoenixin protein expression after LPS injection in several nuclei, possibly resulting from a reduced expression of GPR173, thereby stimulating the upregulation of phoenixin. Another possible explanation could be a physiological expression of phoenixin as an independent neuroprotective agent induced by LPS-induced stress. This theory is supported by a recent publication showing neuroprotective effects of phoenixin-20 via the sirtuin-1 (SIRT-1) pathway [32]. It could be speculated that phoenixin is expressed as part of an anti-inflammatory neuroprotective pathway employing SIRT-1 signaling (Figure 9).

Increased activity in the NTS after LPS injection is a well-established reaction [33], with increased electrical discharge in efferent neurons of the NTS in rats after LPS injection, pointing towards an involvement in the autonomic stress response [34]. The participation of the NTS and the vagus nerve in the efferent inflammation response are well established [35], with sympathetic innervation from the NTS to the spleen possibly modulating splenic immune response [36]. There is also evidence suggesting that peripheral vagal sensory input to the brainstem is necessary for the induction of fever [37]. This activation of the NTS also plays a role in the LPS-induced hypophagia [38]. Our study also observed a significant increase in the number of c-Fos-positive cells in the NTS after IP injection of LPS. The increased expression of phoenixin could be interpreted as a physiological counteraction stimulated by low food intake due to the LPS-induced hypophagia, which was shown before to be—at least in part—mediated by an increase in circulating nucleobindin-2/nesfatin-1 levels [16]. Nesfatin-1 has been shown to play a role in the reaction to several different stressors, such as restraint stress as an emotional stressor [39], as well as immunological stress in response to peripheral inflammatory signals [17]. Since nesfatin-1 and phoenixin were shown to colocalize in several nuclei [40] and have both been shown to be implicated in stress reaction following restraint stress [9,39], a counterbalancing mode of action could be hypothesized.

The DMN is located near the mNTS [23] and is highly interconnected with it [41]. Its parasympathetic neurons [42] influence several aspects, such as energy homeostasis as well as food intake and digestion [43]. Its function is controlled by several brain areas, including the hypothalamus [42]. Interestingly, phoenixin was previously shown to exert anti-inflammatory neuroprotective effects which relied on SIRT-1 [32], a protein also involved in the function of the DMN in energy homeostasis [42], regulating glucose by reducing diet-induced hyperglycemia [44] and increasing peripheral insulin sensitivity [45] while also playing a role in maintaining body weight [46]. Intestinal inflammation, on the other hand, was shown to lead to a decrease in afferent neurons to the DMN and decrease its function [47]. We observed a significant increase in the Fos activity as well as phoenixin immunoreactivity. This coincided with an increased fecal pellet output, suggesting the involvement of the DMN in a faster intestinal passage. The increased phoenixin immunoreactivity could be interpreted as a reaction to apoptotic signaling stemming from peripheral inflammation. This would fall in line with the reported neuroprotective effects of phoenixin employing the SIRT-1 pathway [32], which is vital for normal DMN function [42].

As previously shown [9], the RPa, a nucleus influencing heart rate, body temperature [48,49], modulating pain [50], and antinociception [51,52], is also part of phoenixin’s stress reaction. The 32% increase in phoenixin expression could support our previous theory [9], that increased phoenixin expression in the RPa might be part of a negative feedback loop to control sympathetic activity under conditions of stress, specifically gastrointestinal functions via modulation of the DMN and NTS [53]. The observed increase in phoenixin could also possibly play a role in the observed impairment of gastric emptying under conditions of stress via the DMN [54].

The amygdala, in general, is an important brain site in fear response [55] and is also implicated in the response to LPS [56]. The amygdala remains active longer than other brain nuclei after LPS administration, and its activity correlated with reduced explorative behavior in rodents [56]. Immunohistochemical c-Fos activity in the amygdala after LPS injection has been shown before [57]. We also observed a significant increase in the number of c-Fos cells in the central amygdaloid nucleus after LPS injection, as well as a significant increase in phoenixin immunoreactivity. The amygdala has been shown to increase vasopressin secretion after artificial electrical stimulation [58]. Moreover, the activity of the amygdala is affected by vasopressin and thereby possibly alters fear and anxiety reactions [59], which has been shown to be one of the main effects of phoenixin [5]. Arginine vasopressin neurons in the medial amygdala have also recently been shown to increase hypothalamic vasopressin neuron activity after predator odor stress [60]. Vasopressin neurons in the posterodorsal medial amygdala also influence the reaction of rodents to predator odor stress, leading to a reduction in defensive behavior when these neurons were ablated [61]. This falls in line with our previous observations of phoenixin altering behavior, namely inducing intake of food and water after ICV injection [6] as well as reduced anxiety [5], proposedly mediated through alterations of vasopressin signaling in the amygdala and hypothalamus.

It is unclear whether the observed increase in phoenixin expression is a result of direct interaction of LPS with the respective neurons or if it is secondary to peripheral signaling possibly via the vagus nerve and the NTS, since there are conflicting reports about the effects of LPS on the blood–brain barrier and its ability to penetrate it [62,63,64,65]. A recent report showed differences in the effects of LPS on the blood–brain barrier depending on its region in the brain [63]. Overall, this makes an indirect mode of action most likely, although a small amount of LPS was able to penetrate the blood–brain barrier according to a previous study [62]. Phoenixin might also play a role in the socioemotional behavior in stressful situations, since the neuronal structures studied in this experiment—namely the amygdala—are part of the regions of the brain responsible for reactions to stress [66].

## 5. Conclusions

In conclusion, we showed a significant increase in the number of c-Fos-positive cells and phoenixin expression in the CeM, RPa, SON, DMN, and mNTS after injection of LPS, indicating that several stressors can activate phoenixin signaling but exert a differential response. The present data may further point towards a counterbalancing action of phoenixin and nesfatin-1, e.g., in food intake or gastrointestinal motility and a possible role in the regulation of vasopressin release (Figure 9), although it should be pointed out that this interaction is speculative and must be further investigated. Since the exact mode of action of phoenixin is not yet known, time-course studies will be needed to evaluate whether phoenixin is part of an immediate response or plays a longer-term adaptive role.

## Data Availability

Data can be provided on reasonable request. Please contact the corresponding author.
